# Psychological Resilience, Cardiovascular Disease, and Metabolic Disturbances: A Systematic Review

**DOI:** 10.3389/fpsyg.2022.817298

**Published:** 2022-02-24

**Authors:** Anwal Ghulam, Marialaura Bonaccio, Simona Costanzo, Francesca Bracone, Francesco Gianfagna, Giovanni de Gaetano, Licia Iacoviello

**Affiliations:** ^1^Research Center in Epidemiology and Preventive Medicine, Department of Medicine and Surgery, University of Insubria, Varese, Italy; ^2^Department of Epidemiology and Prevention, Istituto di Ricovero e Cura a Carattere Scientifico (IRCCS) NEUROMED, Pozzilli, Italy; ^3^Mediterranea Cardiocentro, Naples, Italy

**Keywords:** systematic review, cardiovascular disease, psychological resilience, metabolic disturbance, hardiness

## Abstract

**Background:**

Positive psychosocial factors can play an important role in the development of cardiovascular disease (CVD). Among them, psychological resilience (PR) is defined as the capacity of responding positively to stressful events. Our aim was to assess whether PR is associated with CVD or metabolic disturbances through a systematic review.

**Methods:**

We gathered articles from PubMed, Web of Science, PsycInfo, and Google Scholar up to October 28, 2021. We included articles that were in English, were observational, and had PR examined as exposure. The CVD outcomes were either clinical or metabolic outcomes (i.e., dyslipidemia, obesity, metabolic syndrome, hypertension, and diabetes).

**Results:**

Our literature search identified 3,800 studies, of which 17 met the inclusion criteria. Of them, seven were longitudinal and 10 cross-sectional, and 13 were on adults and four on children. The exposure assessment was heterogeneous, i.e., 12 studies used different kinds of self-administered questionnaires and five used interviews with a psychologist. Regarding outcomes, five studies investigated CVD, seven obesity, one metabolic syndrome, two hypertension, four dyslipidemia, and four diabetes. In longitudinal studies, PR was found to have an inverse association with included outcomes in five studies from the Swedish military conscription cohort but had no association with CVD in a study on African-American women and was associated with slower progression of diabetes in a general population. The cross-sectional studies showed that the prevalence of disease was not associated with PR in many cases but the progression of disease was associated with PR.

**Conclusion:**

PR seems to have a possibly favorable association with CVD and metabolic disturbances that differs according to the type of outcome and population. Our study limitations are given by the small number of studies available and the heterogeneity in PR measurement.

**Systematic Review Registration:**

[https://www.crd.york.ac.uk/PROSPERO/display_record.php?RecordID=237109], identifier [CRD42021237109].

## Introduction

Cardiovascular diseases (CVDs) are the leading cause of death globally (Vos et al., 2020). Classical risk factors, such as hypertension, diabetes, dyslipidemia, and obesity, do not fully explain the incidence and prevalence of CVD (Roth et al., 2020). Since CVD and risk factors weigh more on marginalized and economically disadvantaged populations (Schultz et al., 2018), socioeconomic factors (e.g., social conflicts, loneliness, and poverty) and psychological factors (e.g., anxiety, depression, and negative emotions) have been extensively studied for their suspected involvement with CVD. Many studies proved that these factors have a significant independent association with CVD (Everson-Rose and Lewis, 2005).

However, much of this research stemmed from a pathogenetic perspective (from ancient Greek: *patho* = disease, *genesis* = origin) that has been leading medical research for centuries: researchers have long been interested in factors that promote illness. On the other end of the *genesis* spectrum, the *salutogenesis orientation*, a health promotion theory, poses attention on salutary factors, i.e., factors that promote health (Lindström and Eriksson, 2005). We hypothesized that psychological resilience (PR), a positive psychological attribute, could act as a salutary factor and promote cardiovascular health. In fact, a growing body of literature is exploring the relationship between PR and CVD, diabetes, hypertension, and other CVD risk conditions and questioning whether PR can be classified as a protective factor for this array of conditions.

Psychological resilience is the capacity of individuals to cope successfully with significant changes, adversity, or risk (Fletcher and Sarkar, 2013). PR has been conceptualized as a trait, as an outcome, and also as a process. When conceived as a trait, it is understood as a constellation of characteristics that enable individuals to adapt to the circumstances they encounter (Connor and Davidson, 2003); when conceptualized as an outcome, PR is reflected by the positive adaptation after adversity (Fletcher and Sarkar, 2013); when conceptualized as a process, PR is identified as a dynamic process that varies with the time context, age, gender, cultural origin, as well as within an individual subject to different life circumstances (Ungar, 2006). Therefore, individuals can be resilient in some periods of their life or towards certain adversities but not others (Luthar, 2015).

Research exploring the relationship of PR with CVD outcomes is still in its infancy, with some studies pointing to an inverse association between PR, CVD, and metabolic conditions (Topel et al., 2019; Kim et al., 2020; Springfield et al., 2020), while others failed to find any association (Felix et al., 2019; Turkson-Ocran et al., 2020). Thus, we performed a systematic review to summarize the available literature that investigated the relationship between PR and CVD or metabolic outcomes. The systematic review aimed at answering the following research questions:

1. Are psychologically resilient people, i.e., people who adapt better to adversities, less likely to suffer from or develop CVD?
2. Are psychologically resilient people less likely to suffer from or develop metabolic conditions, such as diabetes, dyslipidemia, hypertension, metabolic syndrome, and obesity, compared to less resilient people?
3. Do psychologically resilient people who suffer from a metabolic condition have a slower progression of the disease?
4. Can population subgroups be identified? And if this is the case, does PR act differently in different populations?

## Materials and Methods

The protocol for this systematic review was registered on PROSPERO (CRD42021237109) and is available in full at www.crd.york.ac.uk/PROSPERO/display_record.asp?ID=CRD42 021237109. The systematic review was performed according to the guidelines of the Preferred Reporting Items for Systematic Reviews and Meta-Analyses (PRISMA) group (Page et al., 2020).

### Search Strategy

The systematic search was carried out from inception up to October 28, 2021, for all included databases. The following electronic databases were searched: Medline (provided by PubMed), Web of Science, PsycINFO (provided by EBSCO), and Google Scholar. References from included articles were hand-searched. Results from the bibliographic databases were merged, and duplicates were removed. Keywords used were resilience, resilient, resiliency, psychological resilience, cardiovascular, stroke, strokes, myocardial infarction, CVD, hypertension, blood pressure, systolic, diastolic, diabetes, glucose, glycemia, glycemia, dyslipidemia, hypercholesterolemia, cholesterol, HDL, LDL, triglycerides, lipid profile, obesity, metabolic syndrome, BMI, body mass index, overweight, waist, waist-to-hip, waist to hip, and waist-hip ratio. The full electronic search strategy for all databases and deduplication strategy is presented in the Supplementary Sections 1.1–1.5.

### Study Selection

Studies were included if: (a) the exposure was any assessment of PR; (b) the outcomes were any CVD or the following metabolic conditions: hypertension, dyslipidemia, diabetes, obesity, and metabolic syndrome; (c) study design was observational, cross-sectional, or longitudinal; and (d) language was English. No time restrictions were placed on the search strategy, and the language restriction was chosen due to the impossibility for the authors to translate from the original language. The eligibility assessment was performed by two reviewers (AG and MB), and disagreements between reviewers were resolved by a third reviewer (SC).

### Data Extraction and Quality Assessment

Data extraction was carried out with an excel sheet based on the Cochrane Consumers and Communication Review Group’s data extraction template; it was pilot tested on three included articles and adjusted accordingly. Subsequently, data were extracted by one author (AG) and double-checked for accuracy by another author (MB). The following informations were retrieved: first author, publication year, country, study design, population ethnicity and age (median), recruitment year/s, sample size, follow-up duration (median), PR assessment method and scoring, confounders considered and used, and finally outcomes and outcomes parameters obtained using all statistical models.

Given the conflicting guidance on the risk of bias assessment for observational studies (Siddaway et al., 2019), three different tools, namely, Risk Of Bias In Non-randomized Studies of Interventions (ROBINS-I) (Sterne et al., 2016), Conducting Systematic Reviews and Meta-Analyses of Observational Studies of Etiology (COSMOS-E) guiding questions (Dekkers et al., 2019), and JBI assessment tools (Moola et al., 2020), were pilot tested on two longitudinal studies and two cross-sectional studies. After time and feasibility considerations, COSMOS-E guiding questions, and JBI assessment tools were used in combination. This granted a relatively complete overview and the assessment of the three domains of bias possible in observational studies, namely, confounding, selection, and information bias. COSMOS-E allowed for a better understanding of confounding risk, and JBI allowed for a better assessment of exposure’s measurement quality. In line with what was suggested by the COSMOS-E guide authors, we decided to use a three-level qualitative scale (i.e., high, moderate, and low risks) to assign risk of bias, and for each quality assignment, we presented a rationale with specific references to the study.

## Results

### Search Results

**FIGURE 1:**
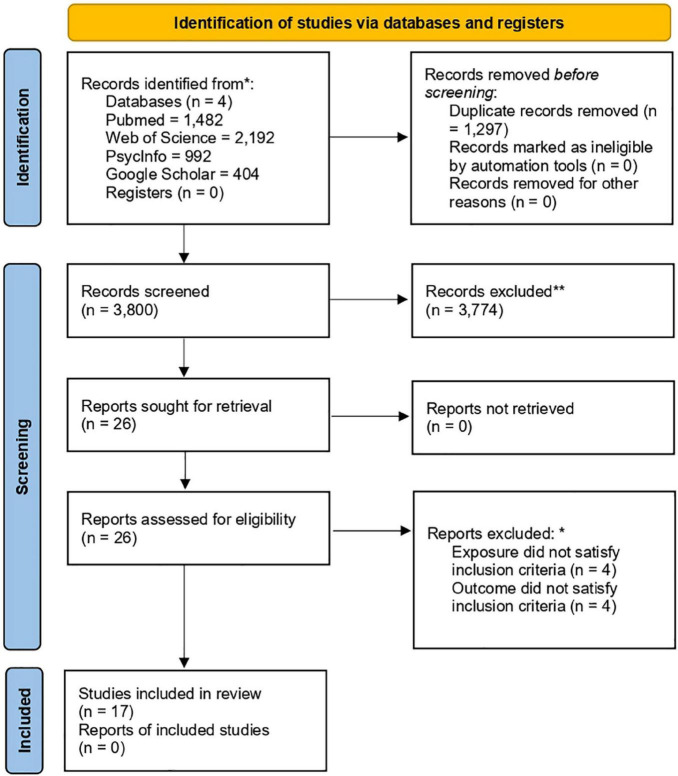
Preferred Reporting Items for Systematic Reviews and Meta-Analyses (PRISMA) flow diagram. *^,^ **More information on excluded articles is present in Supplementary Table 7.

The search of the databases retrieved 3,800 citations after deduplication. Following the screening of the title and abstract, a total of 26 articles were examined in full of which 17 were considered eligible for inclusion (Yi et al., 2008; DeNisco, 2011, Stewart-Knox et al., 2012; Kallem, 2013; Bergh et al., 2014, 2015; Frisillo Vander Veen, 2015; Yi-Frazier et al., 2015; Bartone et al., 2016; Crump et al., 2016a,b; Robertson et al., 2017; Bonaccio et al., 2018; Foster and Weinstein, 2019; Lehrer et al., 2020; Doi et al., 2021). Figure 1 (PRISMA 2020 flow diagram) shows the history of primary studies selection. Even though most studies were at high or moderate risk of bias, we decided to include all of them, since all scored similarly, and it is suggested that risk of bias assessments for observational studies should not be a deterrent to their inclusion (Dekkers et al., 2019). Results are displayed in the Supplementary Tables 1–6. The exclusion of some studies was lengthily debated; therefore, we created a table with characteristics of excluded articles that can be found in the Supplementary Table 7.

### Characteristics of the Included Studies

Characteristics of the studies defined according to the population, intervention/exposure, control, outcome, study type (PICOS) framework were the following: four studies were on children (0–18 years old), five on young adults (18–20 years old), and eight on adults (18 years and above); three recruited African-Americans, five young Swedish men, one Japanese children, one military college attendees in the United States, one black immigrants in the United States, and the remaining six mixed populations from the United States and Europe. The sample size ranged from 50 to over 1,500,000 subjects. PR was measured through self-administered resilience questionnaires in 12 studies and through interviews with a trained psychologist in five studies (details on different assessments are shown in Tables 1, 2). Outcomes were obtained from hospital registers in six studies, were self-reported in three studies, and assessed by a psychologist in the remaining eight studies. Four studies investigated the incidence of CVD (i.e., three studied stroke, heart failure, and coronary heart disease, and one included all CVD), one investigated the prevalence of CVD, one investigated the incidence of hypertension and one prevalence of hypertension, two assessed lipid profiles, one assessed the prevalence of dyslipidemia, one assessed the incidence of diabetes, three assessed HbA1c in diabetic patients, one assessed the prevalence of diabetes, six investigated the prevalence of obesity, and one assessed the prevalence of metabolic syndrome. Included studies were either longitudinal (*n* = 7) or cross-sectional (*n* = 10). A visual description of the characteristics according to the PICOS framework is presented in the Supplementary Tables 8, 9.

**TABLE 1:** Psychological resilience (PR) and cardiovascular disease (CVD) outcomes.

References	Country	Study design	Population (% of men)	Age	PR assessment	Outcome (length of follow-up)	Adjustment	Results
Bergh et al. (2014)	Sweden	Longitudinal	237,879 eligible men for military conscription (100%)	18–20 years	Semi-structured interview	Stroke (23 years)	Birth year, region, parental SEI, and household crowding. Health in adolescence, cognitive function, diastolic and systolic blood pressure, and CVD diagnosis at conscription. Physical fitness in adolescence, physical working capacity and BMI.	HR = 1.16 (95% CI 1.04–1.29) for low vs. high PR, HR adjusted for all covariates.
Bergh et al. (2015)	Sweden	Longitudinal	237,980 eligible men for military conscription (100%)	18–20 years	Semi-structured interview	Coronary Heart Disease (23 years)	Birth year, region, parental SEI, and household crowding. Health in adolescence, cognitive function, diastolic and systolic blood pressure, and CVD diagnosis at conscription. Physical fitness in adolescence, physical working capacity, and BMI.	HR = 1.17 (95% CI 1.10–1.25) for low vs. high PR
Robertson et al. (2017)	Sweden	Longitudinal	1,784,450 eligible men for military conscription (100%)	18–20 years	Semi-structured interview	Early Heart Failure (46 years)	Birth year, region, parental SEI and household crowding, year of military conscription examination, neighborhood SES. Health in adolescence, cognitive function, diastolic and systolic blood pressure, CVD diagnosis at baseline comorbidities at baseline (hypertension, diabetes, congenital heart disease). Diagnosis of nonpsychotic mental disorders. Physical fitness in adolescence, physical working capacity and BMI.	HR = 1.41 (95% CI 1.30–1.53) for low vs. high PR
Felix et al. (2019)	United States	Longitudinal	2,765 postmenopausal African-American women (0%)	79.5 years (SD, 6.6 years; range, 64.6–96.3 years)	Brief resilience scale—modified (three items)	Coronary heart disease, revascularization procedure, carotid artery disease, peripheral artery disease, stroke/transient ischemic attack, heart failure, and CVD-related death (12.5 years)	Social strain, stressful life events, demographics, medical and reproductive history, use of postmenopausal hormone, physical activity, smoking, alcohol, diet, BMI	HR = 0.95 (95% CI 0.63–1.42) (*p* = 0.66) lowest PR quartile vs. highest
Bonaccio et al. (2018)	Italy	Cross-sectional	10,821 subjects from the general population (50.5%)	52.7 ± 10.8	Connor-Davidson Resilience Scale (25 items)	Prevalence of CVD	Age, sex	Prevalence of CVD in high vs. low PR groups = 3.5% vs. 4.7% (*p*-value = 0.011)

**TABLE 2:** Psychological resilience (PR) and metabolic outcomes.

References	Country	Study design	N of subjects (% of men)	Age	PR assessment	Outcome (length of follow-up)	Adjustment	Results
**Hypertension**								
Crump et al. (2016a)	Sweden	Longitudinal	1,547,182 eligible men for military conscription (100%)	18–20 years	Semi-structured interview	Hypertension (43 years)	Birth year, region, parental SEI and household crowding, year of military conscription examination, neighborhood SES. Health in adolescence, cognitive function, diastolic and systolic blood pressure and CVD diagnosis at conscription. Physical fitness in adolescence, physical working capacity and BMI. Type 2 diabetes, family history of hypertension.	HR = 1.43 (95% CI 1.40–1.46) for low vs. high PR
Bonaccio et al. (2018)	Italy	Cross-sectional	10,812 subjects from the general population	(52.7 ± 10.8)	Connor-Davidson Resilience Scale (25 items)	Hypertension	Age, sex	Prevalence of hypertension in high vs. low PR groups = 48% vs. 52.9% (*p*-value = 0.0009)
**Blood lipids**								
Bartone et al. (2016)	United States	Cross-sectional	338 military and civilian population (78%)	44.1 ± 4.5 years	Dispositional Resilience Scale (DRS-15)	Blood lipids	Age, sex	HDL: β = 0.14 (*p*-value < 0.01) Total cholesterol/HDL ratio: β = −0.10 (*p*-value < 0.06)
Bonaccio et al. (2018)	Italy	Cross-sectional	10,821 subjects from the general population (50.5%)	(52.7 ± 10.8)	Connor-Davidson Resilience Scale (25 items)	Hypercholesterolaemia	Age, sex	Prevalence of hypercholesterolaemia in high vs. low PR groups = 31.1% vs. 29.7% (*p*-value = 0.10)
Doi et al. (2021)	Japan	Cross-sectional	1,043 children from elementary and junior high school (47.3%)	13–17 years	Child’s Resilience Coping Scale (eight items)	Blood lipids	Sex, age, household income, maternal and paternal age, diet, physical activity, bedtime difference between school and holiday, child’s self-esteem, parental weight and height, history of diabetes and CVD, mental health	For increase in PR LDL cholesterol: β = −1.26 (95% CI −2.39 to −0.14) HDL cholesterol: β = 0.68 (95% CI −0.68 to 1.82).
**Diabetes**								
Yi et al. (2008)	United States	Longitudinal	145 individuals with diabetes of which 63% type 2 (43%)	49.9 ± 15.1 years	Structural equation modelling. Components were optimism, self-esteem, self-efficacy and self-mastery.	Levels of Hba1c (1 year)	Hba1c levels at baseline	For increase in PR β = −0.39 (*p*-value < 0.01)
Crump et al. (2016b)	Sweden	Longitudinal	1,534,425 eligible men for military conscription (100%)	18–20 years	Semi-structured interview	Type 2 diabetes (25 years)	Birth year, region, parental SEI and household crowding, year of military conscription examination, neighborhood SES. Health in adolescence, cognitive function, diastolic and systolic blood pressure and CVD diagnosis at conscription. Physical fitness in adolescence physical working capacity and BMI. Type 2 diabetes, family history of hypertension, anxiety and depression.	HR = 1.51 (95% CI 1.46–1.57, *p* < 0.0001) for low vs. high PR.
DeNisco (2011)	United States	Cross-sectional	71 women with diabetes (0%)	55 ± 11.1 years	Wagnild and Young Resilience Scale (25 items)	Levels of Hba1c	Ethnicity, gender, age, educational level, marital status, annual income, medical insurance, household status, employment status. Duration of diabetes, treatment medications and chronic illness.	For increase in PR Pearson’s correlation coefficient = –0.350 (*p*-value = 0.003)
Yi-Frazier et al. (2015)	United States	Cross-sectional	50 adolescents with Type 1 diabetes (48%)	13–18 years	Structural equation modelling. Components were optimism, self-esteem and Self-efficacy	Levels of Hba1c	None	Pearson’s correlation coefficient = −0.15 (*p*-value > 0.05)
Bonaccio et al. (2018)	Italy	Cross-sectional	10,812 subjects from the general population (50.5%)	(52.7 ± 10.8)	Connor-Davidson Resilience Scale (25 items)	Diabetes	Age, sex	Prevalence of diabetes in high vs. low PR groups = 7.6% vs. 8.7% (*p*-value = 0.27)
**Anthropometric measurements**								
Stewart-Knox et al. (2012)	Portugal and United Kingdom (UK)	Cross-sectional	1,182 British from the general population (51%) 540 Portuguese from the general population (46.8%)	43–93 years	Wagnild and Young Resilience Scale (RS11)-modified (11 items)	BMI Waist circumference (WC)	Demographics, physical activity, diet, life events, psychological wellbeing (i.e., mood, hopelessness, and perceived stress).	For increase in PR United Kingdom participants BMI: β -0.05 (*p*-value > 0.05) WC: β -0.11 (*p*-value = 0.04) Portuguese participants: BMI: β -0.25 (*p*-value = 0.05) WC: β -0.02 (*p*-value > 0.05)
Kallem (2013)	United States	Cross-sectional	1,523 children eligible for free or reduced price school lunch (46.2%)	9–15 years	Shift and persist modified questionnaire (five items)	BMI	Diet, physical activity, socioeconomic status	β = 0.09 (*p*-value = 0.23)
Frisillo Vander Veen (2015)	United States	Cross-sectional	55 Black immigrants recruited through a web-based survey (41%)	38.0 ± 13.4 years	Connor-Davidson Resilience Scale (25 items)	Obesity health risk through the 20-item Weight-Related Symptom Measure (WRSM) Obesity composite score (BMI + perceived weight + WC)	Age, gender, employment status, marital status, income, level of education, born in United States or not, generation of immigration status, native country	WRSM: β = −0.52 (*p*-value = 0.001) Obesity score: β = −0.175 (*p*-value = 0.244)
Bartone et al. (2016)	United States	Cross-sectional	338 military and civilian population (78%)	44.1 ± 4.5 years	Dispositional Resilience Scale (DRS-15)	BMI	Age, sex	β = −0.14 (*p*-value > 0.01)
Bonaccio et al. (2018)	Italy	Cross-sectional	10,821 subjects from the general population (50.5%)	(52.7 ± 10.8)	Connor-Davidson Resilience Scale (25 items)	BMI Abdominal obesity	Age, sex	Prevalence of obesity (BMI ≥ 30) in high vs. low PR groups = 26.8% vs. 26.8% (*p*-value = 0.45) Prevalence of abdominal obesity in high vs. low PR groups = 70.8% vs. 71.9% (*p*-value = 0.22)
Foster and Weinstein (2019)	United States	Cross-sectional	24,405 children	10–17 years	Resilience scale developed in the frame of the National Survey of Children’s Health (NSCH) and validated by specific questionnaire development lab	BMI	Age, gender, race/ethnicity, insurance status and parental education. Physical activity, environmental stressors, maternal and paternal physical health status	<100% federal poverty line (FPL), OR = 0.63 (95% CI 0.36–1.11) 100–199% FPL, OR = 1.01 (95% CI 0.64–1.59) 200–399% FPL, OR = 1.07 (95% CI 0.79–1.44) >400% FPL, OR = 0.76 (95% CI 0.56–1.05)
**Metabolic syndrome**								
Lehrer et al. (2020)	United States	Cross-sectional	228 subjects mostly university employees (32%)	45 years	Brief Resilience Scale (six items)	Metabolic syndrome severity*	Physical activity, household income, psychological wellbeing (i.e., perceived stress, emotional stability), medication history, hair care (bleach, coloring, conditioner).	For increase in PR β = -0.227 (*p*-value = 0.014)

*Metabolic syndrome severity score calculated from blood lipids, glycemia, ethnicity, BMI, WC, and blood pressure.

*Abbreviations: PR, psychological resilience; HR, hazard ratios; OR, odds ratio; SEI, socioeconomic index; BMI, body mass index; CVD, cardiovascular disease; SES, socioeconomic status; SD, standard deviation; HDL, high-density lipoprotein; LDL, low-density lipoprotein; HbA1c, glycated hemoglobin; WC, waist circumference; WRSM, Weight-Related Symptom Measure.*

A meta-analysis was not performed because of the heterogeneity among included studies: five out of the seven longitudinal studies were based on the same cohort, PR was assessed in different and not comparable ways, and cross-sectional studies were very different in design. In the following paragraphs, studies are described based on our research questions, and a visual description of the main findings is presented in Tables 1, 2.

### Research Questions

#### Question 1: Are Psychologically Resilient People, i.e., People Who Adapt Better to Adversities, Less Likely to Suffer From or Develop Cardiovascular Diseases?

A total of five studies assessed CVD as an outcome, of these, four found an association between PR and CVD, one did not. Three studies from the Swedish military conscription cohort showed that a low PR was associated with a higher risk of CVD. More specifically, the lower tertile of PR score, as compared with the higher tertile, was associated with: (1) increased stroke risk (hazard ratio [HR] = 1.16; 95% CI 1.04–1.29, multivariable model, *n* = 237,879) (Bergh et al., 2014), (2) coronary heart disorders (HR = 1.17; 95% CI 1.10–1.25, multivariable model, *n* = 237,980) (Bergh et al., 2015), and (3) heart failure (HR = 1.41; 95% CI 1.30–1.53, multivariable model, *n* = 1,784,450) (Robertson et al., 2017). In contrast, a longitudinal analysis (Felix et al., 2019) performed in a cohort of 2,765 postmenopausal African-American women showed that PR score was not associated with CVD incidence: the lowest PR quartile compared with the highest showed HR = 0.95 (95% CI 0.63–1.42, *p* = 0.66, multivariable model). Finally, a cross-sectional study from an Italian cohort of 10,821 men and women showed a lower prevalence of CVD in the group with the highest PR compared with the lowest (3.5% vs. 4.7%, *p* < 0.011, in a model adjusted for sex and age) (Bonaccio et al., 2018). Results are displayed in Table 1.

#### Question 2: Are Psychologically Resilient People Less Likely to Suffer From or Develop Metabolic Conditions, Such as Hypertension, Dyslipidemia, Diabetes, Obesity, and Metabolic Syndrome, Compared to Less Resilient People?

Hypertension was assessed in two studies, dyslipidemia in three, diabetes in four, obesity in six, and metabolic syndrome in one. The incidence of hypertension was studied in the Swedish military conscription cohort (*n* = 1,547,182), and results showed that the lower tertile of PR compared with the higher was associated with an increased risk of hypertension (HR = 1.43; 95% CI 1.40–1.46, multivariable model) (Crump et al., 2016a). The prevalence of hypertension was studied in an Italian cohort from the general population (10.821) as self-reported antihypertensive medication use. Results showed a lower frequency of hypertension in the higher PR group as compared with the lower (48% vs. 52.9%; *p*-value = 0.0009, in a model adjusted for sex and age) (Bonaccio et al., 2018).

The prevalence of dyslipidemia was evaluated in three studies, of which two used measures of blood lipids, whereas one used the positive history of lipid-lowering drugs. More specifically (1) in a cohort of 1,043 Japanese school children, results showed that an increase in PR score was associated with a decrease in low-density lipoprotein (LDL) levels (β = −1.26, 95% CI = −2.39 to −0.14, multivariable model) but not with an increase in high-density lipoprotein (HDL) (Bergh et al., 2014); (2) in a cohort of 338 American military college attendees, the increase in PR score was not associated with increased total cholesterol/HDL ratio (β = −0.10, *p*-value < 0.06 in a model adjusted for age and sex) (Moola et al., 2020); (3) in a cohort of 10,821 Italian participants from the Moli-sani study, PR level was not associated with the use of lipid-lowering drugs, in a model adjusted for sex and age (Bartone et al., 2016).

The incidence of diabetes was studied in the Swedish military conscription cohort (*n* = 1,534,425), and results showed that the lower tertile of PR compared with the higher was associated with an increased risk of diabetes (HR = 1.51; 95% CI 1.46–1.57, *p* < 0.0001, multivariable model) (Crump et al., 2016b). The prevalence of diabetes was studied in an Italian cohort as self-reported use of medications for diabetes, and no association was found (Bonaccio et al., 2018).

The prevalence of obesity was studied in seven cohorts that had as outcomes anthropometric measures [i.e., body mass index (BMI) and waist circumference (WC)], and of these, three reported an inverse association. More specifically, (1) a British cohort (*n* = 1,182) from the general population showed that PR score was not associated with BMI, but it was inversely associated with WC (β = -0.11, *p*-value = 0.04, multivariable adjusted model); in contrast, (2) in a general population from Portugal (*n* = 540), PR score was inversely associated with BMI (β = -0.25, *p*-value = 0.05, multivariable adjusted model) but not with WC (Stewart-Knox et al., 2012); and (3) in a cohort of 338 American military college attendees, PR score was inversely associated with BMI (β = -0.14, *p*-value < 0.01 in a model adjusted for age and sex) (Bartone et al., 2016). Other studies did not report any association between PR and the prevalence of obesity (Bergh et al., 2015; Frisillo Vander Veen, 2015; Bonaccio et al., 2018; Foster and Weinstein, 2019).

The prevalence of metabolic syndrome was studied in one cohort of 228 Americans recruited from the general population, and results showed that PR score was inversely associated with metabolic syndrome severity (β = -0.227, *p* = 0.014, multivariable adjusted model) (Lehrer et al., 2020). Results are displayed in Table 2.

#### Question 3: Do Psychologically Resilient People Who Suffer From a Metabolic Condition Have a Slower Progression of the Disease?

Studies that investigated disease progression were performed in diabetic (*n* = 3) and obese (*n* = 1) patients. Three studies enrolled diabetic patients of which two measured their HbA1c level only at the baseline and one also after 1 year of follow-up. Two studies reported an inverse association between PR and HbA1c, while one found no association. More specifically, (1) a cross-sectional study in 71 African-American diabetic women found an inverse association between PR score and HbA1c levels [R = −0.350 (*p*-value = 0.003 multivariable adjusted model)] (DeNisco, 2011); (2) a longitudinal study comparing PR and HbA1c at a year distance found that an increase in PR score was associated with lower HbA1c in a cohort of 145 diabetic patients (β = −0.39, *p*-value < 0.01 in a model adjusted for baseline HbA1c levels) (Yi et al., 2008); (3) a cross-sectional study among 50 teenagers with type I diabetes did not find any association between PR and HbA1c levels (Pearson’s correlation coefficient = −0.15; *p*-value > 0.05 in the crude model) (Yi-Frazier et al., 2015).

One study investigated obesity progression through measures of weight-related health symptoms, among 55 black American immigrants and found that although PR was not associated with prevalence of obesity, subjects with a high PR score were less likely to suffer from weight-related symptoms (β = -0.518, *p* = 0.001) (Frisillo Vander Veen, 2015).

#### Question 4: Can Subgroups of Population Be Identified? and If This Is the Case, Does Psychological Resilience Act Differently in Different Populations?

We categorized populations from the included studies into four subgroups, namely, general populations, vulnerable populations, pediatric populations, and Swedish military conscript cohorts. The subgroup of general populations included four cohorts of adult individuals of both genders from the United States and Europe. More specifically, (1) the British (*n* = 1,182) and Portuguese (*n* = 540) general population showed that PR score was inversely associated with BMI in Portuguese participants but not in British, and PR score was inversely associated with WC in British participants but not in Portuguese (Stewart-Knox et al., 2012); (2) the Italian general population (*n* = 10,821) (Bonaccio et al., 2018) showed that PR was inversely associated with self-reported use of antihypertensive drugs and the presence of CVD disease but not with BMI, self-reported use of lipid-lowering drugs, diabetes medications; (3) the American diabetic patient cohort (*n* = 145) (Yi et al., 2008) showed that PR was inversely associated with HbA1c level at a year’s distance; and (4) the general American population (*n* = 228) (Lehrer et al., 2020) showed that PR was inversely associated with metabolic syndrome severity. The results show that in general population, in the majority of cases PR was inversely associated with outcomes.

The subgroup of vulnerable populations was composed of four cohorts. In the cohort of African-American women (*n* = 2,765) (Felix et al., 2019), no association between PR and CVD incidence was reported; in another cohort of diabetic African-American women (*n* = 71), an inverse association between PR and HbA1c was found (DeNisco, 2011); in the cohort of American children (*n* = 1,523) composed by 85% of Latino and African-American ethnicity eligible for free or reduced-price lunch, no association between PR and BMI (Kallem, 2013) was documented. Finally, the analysis on African immigrants in the United States (*n* = 55) revealed no association between PR and prevalence of BMI but an inverse association with disease progression (Frisillo Vander Veen, 2015). Overall, PR was not associated with disease incidence or prevalence in vulnerable populations, but it was inversely associated with disease progression.

The subgroup of pediatric population was composed of four cohorts. The Japanese pediatric cohort (*n* = 1,043) showed that high PR was associated with lower LDL levels (Doi et al., 2021). The general American pediatric cohort (*n* = 24,405) showed that PR was no longer associated with BMI after adjustment for the parental physical activity level (Foster and Weinstein, 2019); the cohort of mostly African and Latino children also included in vulnerable populations showed no association between PR and BMI (Kallem, 2013). Finally, the cohort of patients with type 1 diabetes (*n* = 50) showed that PR was not associated with HbA1c levels (Yi-Frazier et al., 2015). In summary, PR in pediatric cohorts was inversely associated with blood lipid profile, but it was not associated with obesity prevalence and type 1 diabetes progression.

The Swedish military conscription cohort deserves its own subgroup because studies are all longitudinal, all in male subjects and include the biggest sample size ranging from 237,879 (Bergh et al., 2015) to 1,784,450 (Robertson et al., 2017). All five studies showed that low PR was predictive of a higher risk of diseases (results have been presented above). In the same subgroup, we included the United States military college attendees (*n* = 338) that showed that PR was inversely associated with BMI but not with cholesterol/HDL ratio (Bartone et al., 2016). Overall, in military populations, an increase in PR was inversely associated with the incidence of disease but not with lipid profile.

## Discussion

This systematic review synthesizes results from observational studies on adults and children/adolescents that investigated the association of PR with CVD and metabolic disturbances. Results show mostly inverse associations between PR and CVD: four out of five studies found PR to be inversely associated with CVD; of these four, three were longitudinal and all drew from the same Swedish military conscription registry, and one was a cross-sectional study that found that higher PR was associated with lower CVD prevalence. One longitudinal study did not find an association between PR and CVD. Higher PR was also found to be inversely associated with hypertension in both studies that tested this association. One study out of three that had dyslipidemia as an outcome found an inverse association with PR, as well as three studies out of five that assessed diabetes found that PR was inversely associated with the outcome. Three out of seven studies that tested PR association with anthropometric measures found an inverse association with PR, as well as one study assessing metabolic syndrome.

It is important to put these results into a broader context and discuss in greater depth some of the reasons behind the discrepancy of results and the underlying heterogeneity that makes the comparison challenging.

Part of this challenge has its origins in the operationalization of PR, i.e., the transformation of the theoretical definition in a measurable object. PR defined as a positive adaptation to adversity can change according to the culture because the very meaning of adversity and positive adaptation is informed by that specific culture (Ungar, 2006). Furthermore, PR could be a trait, a dynamic process, and even an outcome. By being dynamic, PR levels can shift across lifespan, or individuals can be more resilient to some adversities and less to others (Fletcher and Sarkar, 2013). These two aspects manifest themselves in the selected studies in the following ways: (1) longitudinal studies may be biased because the assessment of PR happened time before the outcome occurred, and given the dynamicity of PR, the levels of PR at the outcome occurrence might have changed since baseline; (2) included studies recruited subjects from different ethnic backgrounds, but PR scales used were not specific to different cultural origins. Keeping these limitations in mind, we discussed results from two perspectives: outcome nature (i.e., incidence, prevalence, or progression) and population diversity.

### Outcome Nature and Psychological Resilience

Guided by our research questions, we analyzed results based on the nature of the outcomes and classified included studies as incidence (i.e., longitudinal studies), prevalence (i.e., cross-sectional studies), or progression studies (i.e., one longitudinal and the rest are cross-sectional studies). On a total of six longitudinal studies that investigated incidence, five derived their data from the Swedish military conscription registers and showed that a lower PR was consistently associated with an increased risk of incidence of stroke, coronary heart disease, heart failure, diabetes type 2, and hypertension. One longitudinal study recruited African-American women and found no association between PR level and CVD risk. Both cohorts were studied with a detailed covariate adjustment analysis. Hence, PR seems to be inversely associated with outcome in prospective studies; however, it is important to mention that these two populations are extremely different, and results cannot be generalized. PR may well be associated with outcomes in white Swedish young men, and this would not contradict the fact that PR may not be associated with outcomes in African-American women. We argue that these populations have very different social and racial stress burdens: stresses borne by Swedish young men in their own country cannot be compared with stresses burdening the life of African-American women. Black American women notoriously carry a history of discrimination due to their race and gender, which predispose them to a higher risk of diabetes, hypertension, premature birth, and adverse pregnancy outcomes than their white counterparts (IOM, 2003; Giscombé and Lobel, 2005; Black et al., 2015).

The prevalence of metabolic disturbances was studied in nine cohorts that had as outcome BMI, WC, CVD, hypertension, diabetes, and dyslipidemia (through blood lipid profile and lipid-lowering drugs). Results show that PR is not associated with the prevalence of obesity in most cases; it is inversely associated with a better lipid profile in young children but not in adult military servicemen, and in the only study that assessed prevalence of hypertension and CVD, PR was inversely associated with lower hypertension and CVD prevalence. These results are not enough to ascertain or exclude PR association with disease prevalence.

Four articles studied disease progression in relation to PR level, three of these studies refer to diabetic patients and their HbA1c level and one to obesity-related health symptoms. Results from these prospective studies are encouraging: diabetic patients who had higher PR were more likely to have lower HbA1c levels (Yi et al., 2008; DeNisco, 2011). In contrast, young patients with type 1 diabetes reported no association between their PR and HbA1c levels, and this difference may be explained by the very small sample size and by the fact that type 1 diabetes at pediatric age has a different psychological profile that adult type 2 diabetes (Stahl-Pehe et al., 2019). Obesity-related symptoms were significantly lower in higher resilient groups, even though prevalence of obesity is not associated with PR level (Frisillo Vander Veen, 2015).

We concluded that PR is directly associated with better CVD health for young Swedish men, while it is not associated with CVD risk in African-American women. PR is associated with a better lipid profile in Japanese children but not in American military servicemen, and it is not associated with obesity prevalence. Finally, PR is associated with a lower disease progression in diabetic and obese patients.

### Population Diversity and Psychological Resilience

The population analysis showed that patterns of PR association with outcomes differ in subgroups (i.e., general population, vulnerable populations, pediatric population, and Swedish military conscription cohort): more specifically, the vulnerable population subgroup analyses showed that subjects who were already ill and were more resilient had a better disease progression than less resilient subjects, but PR level was not associated with the disease prevalence or incidence rate in any study. The absence of association may be explained by two theories: (1) PR scale used was not adequate to assess the nature of PR in these populations because of the different cultural background and (2) the levels of allostatic load, socioeconomic difficulties, and discrimination in these populations were so high that PR could not counterbalance the negative influence of these adversities on cardiovascular health. It is well-established that minority populations in the United States have worse health outcomes than whites (Usa Census, 2015), have lower socioeconomic status, higher discrimination rates, and suffer more from stress-related diseases (Van Dyke et al., 2020). To assess PR association with outcome in vulnerable populations, it would be advisable to study PR with scales that are culturally reflective and further adjust for perceived stress and life adversities.

The pediatric subgroup analysis showed that PR was associated with lipid profile, but it was not associated with obesity prevalence and diabetes type 1 progression. We concluded that PR is not associated with obesity prevalence in children possibly because the obesogenic environment in pediatric ages is strongly influenced by the caregiver’s attitude and health (Lindsay et al., 2006); it is not surprising that the only study that found an inverse association between PR and obesity lost it after adjusting for the parental physical activity (Foster and Weinstein, 2019); such data may suggest that personal resources of the child are not enough to overcome the risk factors coming from the caregiving environment they are in, and is aligned with the notion that childcare environment is the greatest determinant of PR in developmental age (Masten, 2014). We hypothesized that just as healthy and supportive childcare is associated with increased PR, it may be also inversely associated with obesity; PR could mediate some of the influences of the healthy childcare on obesity. To test this hypothesis, it is recommended to study children and care giver’s PR level, quality of caregiving, and socioeconomic status in one setting.

The Swedish military conscript cohort showed strikingly promising results, with low PR consistently associated with a higher risk of incidence of disease (i.e., CHD, stroke, heart failure, hypertension, and diabetes). All studies were longitudinal and included very large cohorts. It is important to note that PR was measured in a semi-structured psychological interview devised specifically to assess the capacity of the participant to withstand stressors common in military life.

### Strengths and Limitations

This is the first systematic review that explored the association of PR with CVD and metabolic outcomes and the first systematic review that investigated PR as an exposure outside of psychiatric and psychological settings (where PR is studied in relation to psychiatric outcomes). Our strength lies in the systematic search and in-depth population analysis that has become so relevant in the last decades; we acknowledged that there is no one solution to fit all issues, and the same applies to PR that acts differently in different populations, thus interventions and policies should take this into consideration.

Our biggest limitations are that very little literature has investigated PR’s association with diseases incidence, prevalence, or progression, and most of the included studies scored low/moderate in the risk of bias assessment. Thus, our discussion should be read while keeping in mind that PR has been assessed with different self-administered questionnaires that were not always validated and in the Swedish cohort, using interviews with a psychologist; moreover, these tools have not been adapted to cultural differences where appropriate and that each of these tools has been informed by an ever-developing definition of PR. We also fully acknowledged that these limitations are common with most psychological construct assessments. From a methodological standpoint, limitations are given by the fact that we included only English-language articles, we could not access Embase, and we did not perform a meta-analysis because of heterogeneity issues.

To fully understand the impact of PR on health, it would be advisable to involve a multidisciplinary team in future studies that can create and validate the most appropriate assessment tool for that specific population. It is also important to measure the extent of adversity and stress occurring in that period of life that may inform the level of individual resilience.

### Implication of Findings

Finding an association between PR, CVD, and metabolic conditions at risk for CVD is a compelling task. It is well established that populations that carry a history of discrimination, are marginalized, have lower socioeconomic status are also more at risk of CVD, obesity, diabetes, and a range of other dieseases. We assume that these vulnerable populations heavily burdened by disease are also heavily burdened by adversities, where PR would be even more helpful. Even though we do not fully understand PR’s attributes in most non-western cultures, and how each component of PR plays a role in these cultures, discovering an inverse relationship between PR and CVD warrants further investigation of PR in different settings and promoting intervention in vulnerable populations (Chmitorz et al., 2018).

However, it is essential to understand that PR alone can be of little help when the environment is lacking resources. If a subject does not have opportunities to gather resources that help her adapt, measuring adaptability as a function of PR becomes a redundant exercise.

One final point to take into consideration is that PR measurement scales cannot fully capture the complex nature of PR, that has been studied as a trait, a process, or even an outcome (Fletcher and Sarkar, 2013) and that PR manifests only after the occurrence of an adverse event. What we know from the assessment of PR in our studies is the propensity of these populations to adapt positively; however, we have no notion on the adversities that these populations had to face, neither at baseline nor during follow-up period (for longitudinal studies).

Researchers interested in PR association with illness and health should take into consideration the challenge of operationalizing PR outside of psychiatric and psychological settings, assessment of past and present adversities and design better assessment tools that can reflect the understanding of PR among different populations.

## Data Availability Statement

The original contributions presented in the study are included in the article/Supplementary Material, further inquiries can be directed to the corresponding author.

## Author Contributions

AG, MB, SC, and LI designed the research. AG and MB conducted the systematic literature search, performed the quality assessment and the data extraction, and wrote the manuscript. GdG, SC, FG, FB, and LI critically reviewed the manuscript. All authors contributed to reviewing the manuscript and read and approved the final manuscript.

## Conflict of Interest

The authors declare that the research was conducted in the absence of any commercial or financial relationships that could be construed as a potential conflict of interest.

## Publisher’s Note

All claims expressed in this article are solely those of the authors and do not necessarily represent those of their affiliated organizations, or those of the publisher, the editors and the reviewers. Any product that may be evaluated in this article, or claim that may be made by its manufacturer, is not guaranteed or endorsed by the publisher.
