# Supplementary material for: Psychological Resilience, Cardiovascular Disease, and Metabolic Disturbances: A Systematic Review
Source: Front Psychol. 2022 Feb 24;13:817298. doi: 10.3389/fpsyg.2022.817298 (PMC8909142; doi:10.3389/fpsyg.2022.817298)
Supplement: Supplementary file 1 [file Data_Sheet_1.docx]

Supplementary Material

index

[1 Research strategy and strings used 2](#_Toc91539323)

[1.1 Introductory notes 2](#_Toc91539324)

[1.1.1 Concepts: keywords and related terms 2](#_Toc91539325)

[1.1.2 The building blocks of the string 2](#_Toc91539326)

[1.2 Pubmed 3](#_Toc91539327)

[1.3 Web Of Science (core collection) 4](#_Toc91539328)

[1.4 Psychinfo 6](#_Toc91539329)

[1.5 Google Scholar 6](#_Toc91539330)

[2 Risk of bias assessment 7](#_Toc91539331)

[2.1 Part 1. Included longitudinal studies 8](#_Toc91539332)

[2.2 Part 2: included cross-sectional studies 12](#_Toc91539333)

[3 Characteristics of excluded studies 18](#_Toc91539334)

[3.1 References to table S7: 21](#_Toc91539335)

[4 Characteristics of the included studies according to PICOS framework 23](#_Toc91539336)

# Research strategy and strings used

## Introductory notes

### Concepts: keywords and related terms

| Concepts | Related terms |
| --- | --- |
| Psychological Resilience | Resilience, resilient, resiliency, psychological resilience |
| Cardiovascular disease | Cardiovascular, stroke, strokes, myocardial infarction, CVD |
| Hypertension | Hypertension, blood pressure, systolic, diastolic |
| Diabetes | Diabetes, glucose, glycaemia, glycemia |
| Dyslipidaemia | Dyslipidaemia, hypercholesterolemia, cholesterol, HDL, LDL, triglycerides, lipid profile |
| Obesity | obesity, metabolic syndrome, BMI, body mass index, overweight, waist, waist-to-hip, waist to hip, waist hip ratio |

Abbreviation: CVD (cardiovascular disease) LDL (low density lipoprotein) HDL (high density lipoprotein)

### The building blocks of the string

| #1 | resilience or resilient or resiliency or “psychological resilience” |
| --- | --- |
| #2 | cardiovascular or stroke or strokes or "myocardial infarction" or CVD or  hypertension or "blood pressure" or systolic or diastolic or  diabetes or glucose or glycaemia or glycemia or  dyslipidaemia or hypercholesterolemia or cholesterol or HDL or LDL or triglycerides or “lipid profile” or  obesity or “metabolic syndrome” or BMI or "body mass index" or overweight or waist or "waist-to-hip" or "waist to hip" or "waist hip ratio" |

## Pubmed

- Filters: human species & English language
- Research restriction: all fields

|  |  | 28/10/21 |
| --- | --- | --- |
| #3 (#1 and #2) | (resilience or resilient or resiliency or "psychological resilience") and (cardiovascular or stroke or strokes or "myocardial infarction" or CVD hypertension or "blood pressure" or systolic or diastolic or diabetes or glucose or glycaemia or glycemia or dyslipidaemia or hypercholesterolemia or cholesterol or HDL or LDL or triglycerides or "lipid profile" or obesity or "metabolic syndrome" or BMI or "body mass index" or overweight or waist or "waist-to-hip" or "waist to hip" or "waist hip ratio" ) | 1,482 |

## Web Of Science (core collection)

The research in Web of Science has been adapted in 2 ways:

1. #1 has been restricted to “topic” in the field search (instead of “all fields” as in PubMed)

2. there has been a restriction of web of science categories (WC) and Subject areas (SU)

Rationale for adaptation: Resilience is a concept borrowed from Material Science (physics) where it is understood as the ability of a material to absorb energy when it is deformed elastically and release that energy upon unloading. Because of the translatabilty of the concept, it has been widely used widely in different sciences.

Given that Web Of Science is a citation database that includes a variety of science, using an “all field” research and not excluding other sciences that are irrelevant to our research question, has provided very lower specificity. We have decided to use these restrictions after consultation with a field librarian of Insubria University.

- Language: English
- #1* refers to the string #1 researched in “topic” field search restriction.

|  |  | 28/10/21 |
| --- | --- | --- |
| #1 and #2 | TS=((resilience or resilient or resiliency or "psychological resilience") )  AND ALL=((cardiovascular or stroke or strokes or "myocardial infarction" or CVD or hypertension or "blood pressure" or systolic or diastolic or diabetes or glucose or glycaemia or glycemia or dyslipidaemia or hypercholesterolemia or cholesterol or HDL or LDL or triglycerides or "lipid profile" or obesity or "metabolic syndrome" or BMI or "body mass index" or overweight or waist or "waist-to-hip" or "waist to hip" or "waist hip ratio") )  NOT WC=( ECOLOGY OR ENVIRONMENTAL SCIENCES OR MARINE FRESHWATER BIOLOGY OR AGRICULTURE MULTIDISCIPLINARY OR AGRICULTURE DAIRY ANIMAL SCIENCE OR FORESTRY OR FOOD SCIENCE TECHNOLOGY OR FISHERIES OR BIODIVERSITY CONSERVATION OR GEOGRAPHY PHYSICAL OR OCEANOGRAPHY OR PARASITOLOGY OR PLANT SCIENCES OR PHYSICS APPLIED OR CHEMISTRY PHYSICAL OR COMPUTER SCIENCE ARTIFICIAL INTELLIGENCE OR ECONOMICS OR ENTOMOLOGY OR BIOCHEMISTRY MOLECULAR BIOLOGY OR MATERIALS SCIENCE MULTIDISCIPLINARY OR MATHEMATICAL COMPUTATIONAL BIOLOGY OR COMPUTER SCIENCE INFORMATION SYSTEMS OR METEOROLOGY ATMOSPHERIC SCIENCES OR ENGINEERING ELECTRICAL ELECTRONIC OR EDUCATION EDUCATIONAL RESEARCH OR LIMNOLOGY OR ENGINEERING BIOMEDICAL OR EVOLUTIONARY BIOLOGY OR REMOTE SENSING OR CELL BIOLOGY OR CHEMISTRY MULTIDISCIPLINARY OR SOIL SCIENCE OR WATER RESOURCES OR ENGINEERING ENVIRONMENTAL OR COMPUTER SCIENCE INTERDISCIPLINARY APPLICATIONS OR GEOSCIENCES MULTIDISCIPLINARY OR COMPUTER SCIENCE THEORY METHODS OR CONSTRUCTION BUILDING TECHNOLOGY OR TELECOMMUNICATIONS OR ENERGY FUELS OR URBAN STUDIES OR ZOOLOGY OR ENGINEERING CIVIL OR ENVIRONMENTAL STUDIES OR GREEN SUSTAINABLE SCIENCE TECHNOLOGY OR BIOPHYSICS OR AGRONOMY OR SPORT SCIENCES )  NOT SU=( BUSINESS ECONOMICS OR MATHEMATICS OR SCIENCE TECHNOLOGY OTHER TOPICS OR CHEMISTRY OR DEVELOPMENT STUDIES OR GEOCHEMISTRY GEOPHYSICS OR POLYMER SCIENCE OR MECHANICS OR MYCOLOGY OR OPERATIONS RESEARCH MANAGEMENT SCIENCE OR PUBLIC ADMINISTRATION OR VIROLOGY OR MICROBIOLOGY OR AGRICULTURE OR ELECTROCHEMISTRY OR GOVERNMENT LAW OR HISTORY OR MATERIALS SCIENCE OR MEDICAL LABORATORY TECHNOLOGY OR ENGINEERING OR PHYSICS OR PALEONTOLOGY OR BIOTECHNOLOGY APPLIED MICROBIOLOGY OR TRANSPORTATION OR LINGUISTICS OR ZOOLOGY OR RELIGION OR ARCHAEOLOGY OR ARCHITECTURE OR VETERINARY SCIENCES OR COMMUNICATION OR ASIAN STUDIES OR COMPUTER SCIENCE OR AUTOMATION CONTROL SYSTEMS OR CRIMINOLOGY PENOLOGY OR CELL BIOLOGY OR EDUCATION EDUCATIONAL RESEARCH) | 2,192 |
|  | Deduplication conducted after entering DOI collected from Pubmed results in research pane and using “NOT” Boolean operator. | 1,376 |

## Psychinfo

- Research restriction: all fields
- Language: English

|  |  | 28/10/21 |
| --- | --- | --- |
| #6 (#1 and #2) | ( resilience or resilient or resiliency or “psychological resilience” ) AND ( cardiovascular or stroke or strokes or "myocardial infarction" or CVD or hypertension or "blood pressure" or systolic or diastolic or diabetes or glucose or glycaemia or glycemia or dyslipidaemia or hypercholesterolemia or cholesterol or HDL or LDL or triglycerides or “lipid profile” or obesity or “metabolic syndrome” or BMI or "body mass index" or overweight or waist or "waist-to-hip" or "waist to hip" or "waist hip ratio" ) | 992 |
| #7(#6 and not #3) | Deduplication with PMID collected from pubmed results and inserted in research window | 623 |
| #8 (#7 and not #5) | Further deduplication with PMID collected from Web of Science results and inserted in research window | 538 |

## Google Scholar

- restriction: all in title

|  |  | 28/10/2021 |
| --- | --- | --- |
| #1 and #2 | Allintitle: resilience AND (cardiovascular OR stroke OR strokes OR "myocardial infarction" OR CVD OR hypertension OR "blood pressure" OR systolic OR diastolic OR diabetes OR glucose OR glycaemia) | 404 |

Total number of articles to screen after duplicates are removed: 1,482 + 1,376 + 538 +404 = 3,800*

*We were not able to deduplicate results from google scholar because it does not allow a PMID or DOI entrance in the advanced search, so we screened all results from Google Scholar.

# Risk of bias assessment

The assessment is divided in two parts, and contains the following content:

1. Part 1: longitudinal studies
   1. Table S1: JBI critical appraisal checklist for cohort studies
   2. Table S2: COSMOS-E guiding questions
   3. Table S3: qualitative scoring with rationale of included cohort studies
2. Part 2: cross-sectional studies.
   1. Table S4: JBI critical appraisal checklist for analytical cross-sectional studies
   2. Table S5: COSMOS-E guiding questions
   3. Table S6: qualitative scoring with rationale of included cross-sectional studies

Notes on terminology used

- NA = not applicable
- SES = socio economic status

## Part 1. Included longitudinal studies

**Table S1:** JBI critical appraisal checklist for cohort studies

| First author and year | Were the two groups similar and recruited from the same population? | Were the exposures measured similarly to assign people to both exposed and unexposed groups? | Was the exposure measured in a valid and reliable way? | Were confounding factors identified? | Were strategies to deal with confounding factors stated? | Were the groups/participants free of the outcome at the start of the study (or at the moment of exposure)? | Were the outcomes measured in a valid and reliable way? | Was the follow up time reported and sufficient to be long enough for outcomes to occur? | Was follow up complete, and if not, were the reasons to loss to follow up described and explored? | Were strategies to address incomplete follow up utilized? | Was appropriate statistical analysis used? |
| --- | --- | --- | --- | --- | --- | --- | --- | --- | --- | --- | --- |
| Yi 2008 | NA | Yes | No | Partially yes | Partially yes | NA | Yes | Yes | Yes | No | Yes |
| Bergh 2014 | NA | Yes | No | Partially yes | Partially yes | Yes | Yes | Yes | Yes | NA | Yes |
| Bergh 2015 | NA | Yes | No | Partially yes | Partially yes | Yes | Yes | Yes | Yes | NA | Yes |
| Crump 2016a | NA | Yes | No | Partially yes | Partially yes | Yes | Yes | Yes | Yes | NA | Yes |
| Crump 2016b | NA | Yes | No | Partially yes | Partially yes | Yes | Yes | Yes | Yes | NA | Yes |
| Robertson 2017 | NA | Yes | No | Partially yes | Partially yes | Yes | Yes | Yes | Yes | NA | Yes |
| Felix 2019 | NA | Yes | No | Yes | Yes | Yes | Yes | Yes | Yes | Yes | Yes |

**Table s2:** COSMOS-E guiding questions

| First author and year | What are the important variables that might confound the effect of the exposure? * | Were these variables measured with precision and at appropriate points in time? | Did the authors use an appropriate analysis method or design that adjusted for all of the important confounding variables? | Was selection into the study unrelated to both the exposure and outcomes? | Were the reasons for missing data unrelated to the exposure and outcomes? | Were outcome assessors unaware of the exposure status of study participants? | Were the methods of outcome assessment comparable across exposure groups? |
| --- | --- | --- | --- | --- | --- | --- | --- |
| Yi 2008 | Health behaviours (Diet, physical activity), SES, diabetes self-care, some measure of adversity (e.g., perceived stress) | Only diabetes self-care and SES | Only for the confounders identified | Yes | Yes | Yes | Yes |
| Bergh 2014 | Health behaviours (Diet, smoking history, physical activity), SES, some measure of adversity (e.g., perceived stress) | SES and physical activity were controlled for but not at appropriate points in time, CVD history was controlled for | Like above | Yes | Yes | Yes | Yes |
| Bergh 2015 | Like above | Like above | Like above | Yes | Yes | Yes | Yes |
| Crump 2016a | Health behaviours (Diet, smoking history, physical activity), SES, family history of hypertension, some measure of adversity (e.g., perceived stress) | SES and physical activity were controlled for but not at appropriate points in time, family history of hypertension was controlled for. | Like above | Yes | Yes | Yes | Yes |
| Crump 2016b | Health behaviours (Diet, smoking history, physical activity), SES, family history of diabetes, some measure of adversity (e.g., perceived stress) | SES and physical activity were controlled for but not at appropriate points in time, family history of diabetes was controlled for. | Like above | Yes | Yes | Yes | Yes |
| Robertson 2017 | Health behaviours (Diet, smoking history, physical activity), SES, some measure of adversity (e.g., perceived stress) | SES and physical activity were controlled for but not at appropriate points in time, CVD history was controlled for | Like above | Yes | Yes | Yes | Yes |
| Felix 2019 | Like above | Yes | Yes | Yes | Yes | Yes | Yes |

* Note: Given the paucity of evidences on Resilience’s relationship with health and disease risk factors, the suggested confounders are mere hypothesis that we developed using Rothman’s “Epidemiology, an Introduction” as a guide: confounding to occur, requires the following conditions:

1. The confounding factor must be associated with both the risk factor of interest and the outcome.

2. The confounding factor must be distributed unequally among the groups being compared.

3. A confounder cannot be an intermediary step in the causal pathway from the exposure of interest to the outcome of interest.

**Table S3:** qualitative scoring with rationale for included cohort studies

| First author and year | Scoring | Rationale |
| --- | --- | --- |
| Yi 2008 | Moderate risk | 1. Information bias: exposure was created from several questionnaires through structure equation modelling, thus was not validated.  2. confounding: information on diet and physical activity were not retrieved, thus not controlled.  3. selection bias: 34 out of 145 subjects were lost at follow up and strategies to deal with lost at follow up were not reported. An attrition analysis was performed. |
| Bergh 2014 | High risk | 1. information bias: exposure was assessed through semi-structured interview designed to assess resistance to military life stress and was not validated for Resilience assessment.  2. confounding: information on diet and smoking at baseline are not present.  3. confounding: time dependent confounding may be present because of the design: exposure and confounders were assessed at baseline and outcomes were retrieved from hospital registers after more than 20 years of follow up. |
| Bergh 2015 | High risk | Like above |
| Crump 2016a | high risk | Like above |
| Crump 2016b | high risk | Like above |
| Robertson 2017 | High risk | Like above |
| Felix 2019 | Low to Moderate risk | 1. information bias: the exposure was measured through shortened version of a validated assessment tool (Brief Resilience Scale) in a population of African American women which may need a different type of scale that also accounts for social and community support. |

To note: all included longitudinal studies (except Felix et al. 2019), belong to the same cohort of Swedish young men eligible for military conscripts.

## Part 2: included cross-sectional studies

**Table S4:** JBI critical appraisal checklist for analytical cross-sectional studies

| First author and year | Were the criteria for inclusion in the sample clearly defined? | Were the study subjects and the setting described in detail? | Was the exposure measured in a valid and reliable way? | Were objective, standard criteria used for measurement of the condition? | Were confounding factors identified? | Were strategies to deal with confounding factors stated? | Were the outcomes measured in a valid and reliable way? | Was appropriate statistical analysis used? |
| --- | --- | --- | --- | --- | --- | --- | --- | --- |
| DeNisco 2011 | Yes | Yes | Yes | Yes | Partially yes | Partially yes | Yes | Yes |
| Stewart-Knox 2012 | No | Yes | Yes | Yes | Yes | Yes | Yes | Yes |
| Kallem 2013 | No | Yes | No | Yes | Yes | Yes | Yes | Yes |
| Frisillo 2015 | Yes | Yes | Yes | Yes | No | No | No | Yes |
| Yi & Franzier 2015 | Yes | Yes | No | Yes | No | No | Yes | Yes |
| Bartone 2016 | Not sure | Yes | Yes | Yes | No | No | Yes | Yes |
| Bonaccio 2018 | Yes | Yes | Yes | Yes | No | No | Yes | Yes |
| Foster & Weinstein 2019 | Yes | Yes | Not sure | Yes | Yes | Yes | No | Yes |
| Lehrer 2020 | Yes | Yes | Yes | Yes | Partially yes | Partially yes | Yes | Yes |
| Doi 2021 | Yes | Yes | Yes | Yes | Yes | Yes | Yes | Yes |

**Table S5:** COSMOS-E guiding questions

| First author and year | What are the important variables that might confound the effect of the exposure? | Were these variables measured with precision and at appropriate points in time? | Did the authors use an appropriate analysis method or design that adjusted for all of the important confounding variables? | Was selection into the study unrelated to both the exposure and outcomes? | Were the reasons for missing data unrelated to the exposure and outcomes? | Were outcome assessors unaware of the exposure status of study participants? | Were the methods of outcome assessment comparable across exposure groups? |
| --- | --- | --- | --- | --- | --- | --- | --- |
| DeNisco 2011 | Health behaviours (Diet, physical activity), SES, diabetes self-care behaviours, some measure of adversity (e.g., perceived stress) | Not sure | Not sure | Yes | Yes | Yes | Yes |
| Stewart-Knox 2012 | Health behaviours (Diet, physical activity), SES, some measure of adversity (e.g., perceived stress) | Yes | Yes | Yes | Yes | Not sure | Yes |
| Kallem 2013 | Like above | Partially yes | Not sure | Not sure | Yes | Yes | Yes |
| Frisillo 2015 | Like above | No | No | Yes | Yes | Yes | Yes |
| Yi & Franzier 2015 | Health behaviours (Diet, physical activity), SES, diabetes self-care, some measure of adversity (e.g., perceived stress, bullyism) | No | No | No | Yes | Yes | Yes |
| Bartone 2016 | Health behaviours (Diet, physical activity), SES, some measure of adversity (e.g., perceived stress) | No | No | Yes | Yes | Yes | Yes |
| Bonaccio 2018 | Health behaviours (Diet, physical activity), SES, diabetes self-care, family history of hypertension and diabetes, some measure of adversity (e.g., perceived stress) | No | No | Yes | Yes | Yes | Yes |
| Foster & Weinstein 2019 | Health behaviours (Diet, physical activity), SES, parental health behaviours (diet and physical activity), some measure of adversity (e.g., perceived stress, bullyism) | Yes | Yes | Yes | Yes | Yes | Yes |
| Lehrer 2020 | Health behaviours (Diet, physical activity), SES, some measure of adversity (e.g., perceived stress) | Partially yes | Partially yes | Yes | Yes | Yes | Yes |
| Doi 2021 | Health behaviours (Diet, physical activity), SES, parental health behaviours (diet and physical activity), some measure of adversity (e.g., perceived stress, bullyism) | Yes | Yes | Yes | Yes | Yes | Yes |

**Table S6**: qualitative scoring with rationale for included cross-sectional studies

| First author and year | Scoring | Rationale |
| --- | --- | --- |
| DeNisco 2011 | Moderate risk | 1. information bias - some participants needed help in understanding exposure assessment.  2. confounding. Diet and physical activity are investigated but it is not clear whether they are controlled.  3. selection bias, only African American were to be included, but women from other ethnicities are also present in final sample |
| Stewart-Knox 2012 | Low to moderate risk | 1. information bias: it is not clearly stated how questions were asked: was it the interviewer asking questions or was her merely present while participants filled the questionnaires on their own.  2 information bias: assessment of exposure and outcome was done in one session with the presence of the interviewer. |
| Kallem 2013 | Moderate risk | 1. selection bias: inclusion criteria for the study participants did not match the final recruited population: all English-speaking classes from schools belonging to one district were eligible and participants were randomly recruited, but in the recruited population, more than 89% qualify as socio-economically disadvantaged.  2. information bias: the shorter version of the questionnaire is not validated yet |
| Frisillo 2015 | High risk | 1. confounder were not identified for resilience because resilience was not the main exposure.  2. information bias: outcome (weight) was self-reported |
| Yi and Franzier 2015 | High risk | 1. confounding: no confounder was identified  2. information bias: exposure assessment was not validated |
| Bartone 2016 | High risk | 1. selection bias: not clear how they recruited the participants  2: confounding: only BMI, age and sex were controlled for, there was no information on diet and physical activity |
| Bonaccio 2018 | High risk | 1. confounders were not identified because the study had an outcome different from the ones of interest for this systematic review (Mediterranean diet was the outcome) |
| Foster & Weinstein 2019 | Low risk | - |
| Lehrer 2020 | Low to moderate risk | 1. information bias: SES was not accounted for |
| Doi 2021 | Low risk | - |

# Characteristics of excluded studies

Table S7: characteristics of excluded studies

| First author and year | Country | Study design | Population (%men and age) | Exposure | Outcome (length of follow up) | Results | Reason for exclusion |
| --- | --- | --- | --- | --- | --- | --- | --- |
| Gallo et al. 2007[1] | USA | Cross-sectional | 145 Latina women recruited from health clinics along the California- Mexico border. age: 47.07 (SD=8.21) | Resilient resources**:** determined with a composite score made by 4 questionnaires: 1- life orientation test-Revised, 10 items (perceived control) 2- Mastery Scale 7 items (mastery) 3- Rosenberg's self-esteem scale 6 items (self-esteem), 4- Interpersonal support evaluation list 12 items (social support) | Metabolic syndrome assessed with measures of systolic and diastolic blood pressure, waist circumference, HDL, triglycerides Glucose | Resilient resources independently predicted lower Waist circumference delta R2= 0.07, p < .05. | Resilience assessment did not satisfy inclusion criteria because it was not based on a resilience questionnaire or interview. |
| Robinson 2013[2] | USA | Cross-sectional | 200 African American subjects 37% men, age: 43.4 (SD=2) | Dispositional resilience scale | Health risk index score made from beck depression index, Pittsburgh sleep quality index, Paffenbarger physical activity questionnaire, mean arterial pressure, high density lipoprotein, triglycerides fasting glucose and waist circumference | Na | Outcome was not CVD or metabolic condition |
| Hopkins et al. 2015 [3] | USA | Cross-sectional | 677 Australian aboriginal youth and their families, 50.4% men. | A composite variable of psychosocial resilient status, derived by cross-classifying youth by high/low family risk exposure and normal/abnormal psychosocial functioning, resulted in four groups- Resilient, Less Resilient, Expected Good and Vulnerable | Asthma problems (three survey questions developed for the International Study of Asthma and Allergies in Childhood (ISAAC) and lifetime health problems (epilepsy, kidney/renal disease, arthritis/rheumatism, developmental delay, muscular dystrophy, recurring chest, ear and/or skin infections, allergies) | Not relevant | Resilience assessment did not satisfy inclusion criteria because it was not based on a resilience questionnaire or interview. |
| Yi-Franzier et al. 2018 [4] | USA | Cross-sectional | Newly diagnosed type 1 diabetes patient, 59 subjects, 61% men, age: 13.2 (SD=2.1) | 10-item Connor and Davidson | Stress trajectory determined by semiparametric group-based trajectory modeling. | Not relevant | Outcome was not CVD or metabolic outcome |
| Topel et al. 2020 [5] | USA | Cross-sectional | 1,433 blacks from MECA study, 38% men, age: 51.6 (SD=10.1) | 10 item Connor and Davidson scale | To be residents of neighborhood with low vs high risk of CVD diseases | Resilience was not associated with neighborhood cardiovascular risk with OR=0.99 (0.80-1.23) | Outcome was a CVD outcome but referred to the neighborhood level |
| Kim et al. 2020 [6] | USA | Cross-sectional | 389 Black adults, 39% men, age: 53+- 10 years | Connor and Davidson resilience scale 10 items | Life's simple 7 | Higher individual psychosocial resilience was significantly associated with higher LS7 (β=0.38 [0.16–0.59] for each SD) | Outcome was a composite score |
| Tukson et al. 2020 [7] | USA | Cross-sectional | 342 subjects, from the African immigrant health study (39% men), age: 47 (SD=11) | Acculturation | Diabetes, high cholesterol, hypertension, obesity, self-reported smoking, diet quality (physical activity from life's simple 7 was not included) | Not relevant | Exposure was not Resilience |
| Springield et al. 2020 [8] | USA | Cross-sectional | 77,395 black, whites and Latinas from the Women’s Health Initiative study, (age >65) | Brief resilience scale | Cvd protective health behavior made from: diet quality (healthy eating index), smoking status, physical activity, recommended sleep hours per night and alcohol consumption | Not relevant | Outcomes was not CVD or metabolic outcomes |
| Nishimi et al. 2021 [9] | USA | Longitudinal and cross sectional | 3,254 subjects from Midlife in the United States 44% male and 93% white, age: 46.2 +-12.1 | Psychological resilience was defined according to both early psychosocial adversity and adult psychological health (characterized by low distress and high wellbeing) The classification is as follows: favorable psychological functioning with adversity (resilient; adversity-exposed, high psychological health), unfavorable psychological functioning with adversity (nonresilient; adversity-exposed, lower psychological health), unfavorable psychological functioning without adversity (adversity-unexposed, lower psychological health), and favorable psychological functioning without adversity (adversity-unexposed, high psychological health) | self-reported incident cardiometabolic conditions: heart attack, stroke and diabetes 2004-5 and 2013-2014. (Follow up 20 y). Secondary outcomes were biomarkers in 2004-2005 | Among those exposed to adversity, unfavorable versus favorable psychological functioning was associated with 43% higher odds of incident cardiometabolic conditions, OR = 1.43, 95% CI 1.10 to 1.85). | Resilience assessment could not satisfy inclusion criteria because it was not based on a resilience questionnaire or interview. |

## References to table S7:

1. Gallo LC, de los Monteros KE, Ferent V *et al.* Education, psychosocial resources, and metabolic syndrome variables in Latinas. *Ann Behav Med* 2007;**34**:14–25.

2. Robinson SR. ASSESSING THE RELATIONSHIP OF HEALTH LOCUS OF CONTROL, HARDINESS, AND HEALTH RISKS IN AFRICAN AMERICANS. 2013:100.

3. Hopkins KD, Shepherd CCJ, Taylor CL *et al.* Relationships between Psychosocial Resilience and Physical Health Status of Western Australian Urban Aboriginal Youth. *PLoS ONE* 2015;**10**, DOI: 10.1371/journal.pone.0145382.

4. Yi-Frazier JP, Cochrane K, Whitlock K *et al.* Trajectories of Acute Diabetes-Specific Stress in Adolescents With Type 1 Diabetes and Their Caregivers Within the First Year of Diagnosis. *J Pediatr Psychol* 2018;**43**:645–53.

5. Topel ML, Kim JH, Mujahid MS *et al.* Individual Characteristics of Resilience are Associated With Lower‐Than‐Expected Neighborhood Rates of Cardiovascular Disease in Blacks: Results From the Morehouse‐Emory Cardiovascular (MECA) Center for Health Equity Study. *J Am Heart Assoc* 2019;**8**, DOI: 10.1161/JAHA.118.011633.

6. Kim JH, Islam SJ, Topel ML *et al.* Individual Psychosocial Resilience, Neighborhood Context, and Cardiovascular Health in Black Adults. *Circ Cardiovasc Qual Outcomes* 2020, DOI: 10.1161/CIRCOUTCOMES.120.006638.

7. Turkson-Ocran R-AN, Szanton SL, Cooper LA *et al.* Discrimination Is Associated with Elevated Cardiovascular Disease Risk among African Immigrants in the African Immigrant Health Study. *Ethn Dis* **30**:651–60.

8. Springfield S, Qin F, Hedlin H *et al.* Resilience and CVD-protective Health Behaviors in Older Women: Examining Racial and Ethnic Differences in a Cross-Sectional Analysis of the Women’s Health Initiative. *Nutrients* 2020;**12**, DOI: 10.3390/nu12072107.

9. Nishimi KM, Koenen KC, Coull BA *et al.* Psychological resilience predicting cardiometabolic conditions in adulthood in the Midlife in the United States Study. *Proc Natl Acad Sci* 2021;**118**:e2102619118.

# Characteristics of the included studies according to PICOS framework

**Table S7:** summary of characteristics of the included studies with favourable outcome on total outcome %

|  | Characteristics | Category | n | n* | % |
| --- | --- | --- | --- | --- | --- |
| P | Population age | Children (10-18 years) | 4 |  |  |
|  |  | Young adults (18-20 years) | 5 |  |  |
|  |  | Adults (> 20) | 7 |  |  |
|  | Population ethnicity | Black-Americans | 3 |  |  |
|  |  | American of mixed ethnicity | 6 |  |  |
|  |  | European | 7 |  |  |
|  |  | Japanese | 1 |  |  |
| I | Exposure measurement tool | Self-made resilience score | 3 |  |  |
|  |  | Connor and Davidson resilience scale (CD-RISC 25) | 2 |  |  |
|  |  | Wagnild and Young resilience scale (RSS 25) | 1 |  |  |
|  |  | Wagnild and Young resilience scale (RSS 11) | 1 |  |  |
|  |  | Dispositional resilience scale (DRS 15) | 1 |  |  |
|  |  | Brief resilience scale (BRS 6) | 1 |  |  |
|  |  | Brief resilience scale (BRS 3) | 1 |  |  |
|  |  | Child’s resilience coping scale (CRCS 8) | 1 |  |  |
|  |  | Shift and Persist questionnaire | 1 |  |  |
|  |  | Semi-structured Interview | 5 |  |  |
| O | Outcome | CVD | 5 | 4 | 80% |
|  |  | Obesity: BMI, metabolic syndrome | 8 | 4 | 50% |
|  |  | Hypertension | 2 | 2 | 100% |
|  |  | Dyslipidaemia | 3 | 1 | 33% |
|  |  | Diabetes | 5 | 3 | 60% |
| S | Type of study | Longitudinal | 7 |  |  |
|  |  | Cross-sectional | 10 |  |  |

n* is the number of studies that reported a favourable association between psychological resilience and outcome.

% is the percentage of favourable outcomes on totality of studies addressing that outcome.

Note, the totality of outcomes exceeds the total number of studies because some studies investigated multiple outcomes

**Table S8:** characteristics of each included study according to PICOS

| **First author and year** | **population** | | | | | | | | | **exposure** | | | **outcome** | | | | | |
| --- | --- | --- | --- | --- | --- | --- | --- | --- | --- | --- | --- | --- | --- | --- | --- | --- | --- | --- |
|  | gender | | age | | | ethnicity | | | | questionnaire | | interview |  | | | | | |
|  | Male | Female | Adults 18 years and above | Young Adults 18-20 years | Children 0-18 years | African American | North American | European | Asian | Validated | Not validated |  | CVD | obesity | Metabolic syndrome | Hypertension | Dyslipidaemia | Diabetes |
| **Longitudinal studies** | | | | | | | | | | | | | | | | | | |
| DeNisco 2011 |  | 1 | 1 |  |  | 1 |  |  |  | 1 |  |  |  |  |  |  |  | 1 |
| Stewart-Knox 2012 | 1 | 1 | 1 |  |  |  |  | 1 |  | 1 |  |  |  | 1 |  |  |  |  |
| Kallem 2013 | 1 | 1 |  |  | 1 | 1 | 1 |  |  |  | 1 |  |  | 1 |  |  |  |  |
| Frisillo 2015 | 1 | 1 | 1 |  |  |  |  |  |  | 1 |  |  |  | 1 |  |  |  |  |
| Yi-Franzier 2015 | 1 | 1 |  |  | 1 |  | 1 |  |  | 1 |  |  |  |  |  |  |  | 1 |
| Bartone 2016 | 1 | 1 | 1 |  |  |  | 1 |  |  | 1 |  |  |  | 1 |  |  | 1 |  |
| Bonaccio 2018 | 1 | 1 | 1 |  |  |  |  | 1 |  | 1 |  |  | 1 | 1 |  | 1 | 1 | 1 |
| Foster & Weinstein 2019 | 1 | 1 |  |  | 1 |  | 1 |  |  | 1 |  |  |  | 1 |  |  |  |  |
| Lehrer 2020 | 1 | 1 | 1 |  |  |  | 1 |  |  | 1 |  |  |  |  | 1 |  |  |  |
| Doi 2021 | 1 | 1 |  |  | 1 |  |  |  | 1 | 1 |  |  |  |  |  |  | 1 |  |
| **Cross-sectional studies** | | | | | | | | | | | | | | | | | | |
| Yi 2008 | 1 | 1 | 1 |  |  |  | 1 |  |  |  | 1 |  |  |  |  |  |  | 1 |
| Bergh 2014 | 1 |  |  | 1 |  |  |  | 1 |  |  |  | 1 | 1 |  |  |  |  |  |
| Bergh 2014 | 1 |  |  | 1 |  |  |  | 1 |  |  |  | 1 | 1 |  |  |  |  |  |
| Crump 2016a | 1 |  |  | 1 |  |  |  | 1 |  |  |  | 1 |  |  |  | 1 |  |  |
| Crump 2016 b | 1 |  |  | 1 |  |  |  | 1 |  |  |  | 1 |  |  |  |  |  | 1 |
| Robertson 2017 | 1 |  |  | 1 |  |  |  | 1 |  |  |  | 1 | 1 |  |  |  |  |  |
| Felix 2019 |  | 1 | 1 |  |  | 1 |  |  |  | 1 |  |  | 1 |  |  |  |  |  |
